# Tolerance for Nutrient Imbalance in an Intermittently Feeding Herbivorous Cricket, the Wellington Tree Weta

**DOI:** 10.1371/journal.pone.0084641

**Published:** 2013-12-17

**Authors:** Priscilla M. Wehi, David Raubenheimer, Mary Morgan-Richards

**Affiliations:** 1 Ecology Group, Institute of Agriculture and Environment, Massey University, Palmerston North, New Zealand; 2 Institute of Natural Sciences, Massey University, North Shore, Auckland, New Zealand; 3 The Charles Perkins Centre and Faculty of Veterinary Science and School of Biological Sciences, The University of Sydney, New South Wales, Australia; National Institute of Agronomic Research, France

## Abstract

Organisms that regulate nutrient intake have an advantage over those that do not, given that the nutrient composition of any one resource rarely matches optimal nutrient requirements. We used nutritional geometry to model protein and carbohydrate intake and identify an intake target for a sexually dimorphic species, the Wellington tree weta (*Hemideina crassidens*). Despite pronounced sexual dimorphism in this large generalist herbivorous insect, intake targets did not differ by sex. In a series of laboratory experiments, we then investigated whether tree weta demonstrate compensatory responses for enforced periods of imbalanced nutrient intake. Weta pre-fed high or low carbohydrate: protein diets showed large variation in compensatory nutrient intake over short (<48 h) time periods when provided with a choice. Individuals did not strongly defend nutrient targets, although there was some evidence for weak regulation. Many weta tended to select high and low protein foods in a ratio similar to their previously identified nutrient optimum. These results suggest that weta have a wide tolerance to nutritional imbalance, and that the time scale of weta nutrient balancing could lie outside of the short time span tested here. A wide tolerance to imbalance is consistent with the intermittent feeding displayed in the wild by weta and may be important in understanding weta foraging patterns in New Zealand forests.

## Introduction

The nutritional composition of foods rarely matches the nutritional requirements of an organism. Moreover, nutrients in plants that are important for animal growth, such as protein, vary both temporally and spatially, as do characteristics such as digestibility and secondary metabolites [1,2]. The ratio of protein to digestible carbohydrate in plants is thus likely to determine feeding patterns in herbivores, influencing foraging rates and strategies and other aspects of performance [3]. Generalist herbivores tend to be both mobile and selective [4], moving widely to encounter suitable foods and avoid nutrient limitation. They frequently switch between foods, but are likely to eat large amounts of imbalanced foods [5,6]. Nonetheless, many organisms have a demonstrated ability to select combinations of complementary foods that individually are nutritionally imbalanced, but together allow an animal to reach its multidimensional intake target [2,7,8].

Natural selection should favour individuals that can regulate nutrient intake using resource selection strategies such as carnivory or predation (including occasional cannibalism), or differential absorption and utilisation of nutrients 1], [6], [because failure to balance nutrient intake can result in large fitness costs [4,9,10]. This might occur, for example, if intake of a key limiting nutrient is prioritized, and results in a situation where other nutrients are over-consumed. The extent to which an individual overeats one nutrient while under-eating another thus represents a compromise [11]. Experiments which limit key nutrients during short periods of pre-feeding have shown that individuals in a range of species are capable of generating nutrient specific compensatory responses (e.g. cockroaches [12], beetles [13], and spiders [14]).

It has been argued, however, that nutritionally explicit models should consider functional consequences and mechanistic constraints of nutritional decisions [3,15]. For example, where males and females differ in their reproductive strategies, such as the timing and costs of reproductive effort [16,17,18,19], sexually dimorphic optima in nutrient requirements and the allocation of resources might also occur. Nutrient requirements could also differ between the sexes because of different costs associated with somatic maintenance, intra-specific competition, or immune function [20,21,22,23]. Here, we consider nutrient requirements associated with one particular organismal trait, sexually dimorphic weaponry. If the sex-specific resource optima hypothesis is correct, then males and females might demonstrate different nutrient intake targets in late instar stages when sexual weaponry is developing. Alternatively, the sexes could differ in the type and amount of nutrients that are most tightly regulated or prioritised during food selection and hence differ in patterns of compensatory nutrient intake. Characterization of sex-specific nutritional optima thus has clear utility for probing behavioural and physiological mechanisms governing how each sex forages.

We investigated nutrient intake target and consumption in a sexually dimorphic cricket the Wellington tree weta (*Hemideina crassidens*), using the geometric framework for nutrition [24,25]. Tree weta (*Hemideina* spp.) are large bodied Orthopterans that are ecologically important in New Zealand forests, and preferred prey for both native birds and invasive mammals such as rats [26,27,28]. These cryptic foragers are nocturnal and have an adult weight range of approximately 3–6 g). However, whereas females mature at the 10^th^ instar only, males can mature asynchronously at either 8^th^, 9^th^ or 10^th^ instar [29,30]. Instar at maturity reflects male investment in heavy, mandibular weaponry [30], such that weight is a useful proxy for male investment in these sexually selected weapons. Adult weta can survive for at least a year [31] and MMR and PMW (personal observation). 

Most Anostostomatidae crickets tend towards carnivory, but the Wellington tree weta *Hemideina crassidens* is likely a generalist herbivore, similar to other species in this genus [32,33]. Nonetheless, informal reports also indicate occasional carnivory as a foraging strategy [32,34]. In *H. crassidens*, large headed males with elongated mandibles engage in combat to defend cavities with female harems in a polygynandrous mating system [35,36]. The heads of the two sexes are completely indistinguishable prior to the 5^th^ instar, and most dimorphic mandibular development occurs in late instar males immediately prior to sexual maturity, consistent with other Orthopterans [37,38]. It is thus possible that late sub-adult and adult males could have specific nutrient needs based either on the cost of developing or maintaining large mandibles, or costs associated with male-male combat. 

We asked whether *Hemideina crassidens* individuals regulate their nutritional intake to meet the demand for specific nutrient combinations [39]. Using artificial foods, we investigated nutrient intake in these individuals to determine their nutritional targets. We predicted that males would show increased protein demand relative to females if the development and maintenance of male weaponry has high nutrient costs. We then conducted trials to determine whether a period of enforced imbalanced nutrient intake would result in subsequent compensatory feeding, using both artificial foods and foods available to them in their natural environment, over two time scales. We compare our results with data for other insects, to examine the possibility that feeding differences might be related to key differences in life history, and discuss the implications of the data for the understanding of weta foraging patterns and their ecological niche in New Zealand forests [40]. 

## Methods

### Tree weta capture and maintenance

This work did not involve endangered or protected species. Palmerston North City Council supported the research in the Turitea Valley, Manawatū, New Zealand. This site has pine trees (*Pinus radiata*) with a layer of native forest regenerating beneath, and is part of a managed reserve of 3500 ha. A total of 32 *Hemideina crassidens* were collected in March 2011, November 2011 and March 2012 from artificial cavities attached to pine tree trunks in the Turitea Valley, Manawatu, New Zealand. Weta in a range of instars were collected, as is consistent with the life stage asynchrony normally observed in this species. While it was not possible to identify instars precisely, weight and tibia length (size), which are highly correlated in tree weta [32], provide a generally reliable proxy for developmental stage. Individuals were therefore weighed, sexed and then housed in ambient outdoor conditions on site for the artificial feeding trial. For these weta, housing consisted of a 2L plastic container for each individual. Each container was provided with a damp paper towel and a daytime shelter consisting of a hollowed harakeke (*Phormium tenax*) flower stalk. Containers had fine mesh inserted into the lids to allow light and air circulation, and were sprayed every two days with water to ensure moist conditions optimal for weta survival. A separate water source is not required. Examination of frass pellets collected from all individuals confirmed that variation in the proportion of animal material eaten prior to capture was small, with no animal body parts observed in 28 of 32 frass. 

### Nutrient Intake Trial

After three days of acclimation during which individuals were not fed, we provided each individually maintained, wild-caught tree weta with two suboptimal but nutritionally complementary chemically defined foods, as detailed by [41]. One had a high protein/carbohydrate ratio (diet P; 28% protein and 7% carbohydrate) and the other had a low-protein/ carbohydrate ratio (diet C; 28% carbohydrate and 7% protein), but the foods were in other respects identical. Protein was a 3:1:1 ratio of casein, peptone and albumin, and the carbohydrates used were sucrose and dextrin (1:1). Two 12 cm^2^ petri dishes containing approximately 1 g each of the foods were placed 4 cm apart in each container. Each dish of dried food was weighed to the nearest 0.01 mg before being sprayed with water. The relative positions of the dishes were randomised across containers and time periods. Tree weta did not have access to any other foods, but enough moisture was available in the food, and on the sides and floor of the container, to ensure weta were not dehydrated. 

During the experiment each weta was allowed to feed for 24 hours in the first instance, after which both food dishes were removed and replaced with fresh, pre-weighed dishes of food for a further 24 hours. Finally, dishes were left for a further five days, so that each individual was held in captivity for a total of seven days before being returned to the wild. All removed food was dried at 60°C for 48 h and its dry weight then recorded. The weight difference for each dish, before and after consumption, provided an estimate of the amount of each food eaten, and thus the total amount of protein and carbohydrate consumed at intervals of 24 hours, 48 hours, and seven days. Although a minimal amount (<1%) of food was spilled by weta, its damp consistency ensured spillage was almost always adjacent to the dish. Spilled food was returned to the appropriate container for weighing after a further check for colour matching; very little food was unidentifiable. All frass produced during the seven day period was recorded, dried at 50°C for 48 hours and weighed. 

Individuals were deemed to have fasted, and were removed from the analysis if they produced no more than two frass pellets over the seven day experiment and/or their combined food weight difference was less than 100 mg (10%) over seven days. Weta will not eat for a number of days prior to moulting (pers. obs. MMR).

### Influence of nutritional state on subsequent diet choice

A further group of 32 previously untested captive *Hemideina crassidens* were tested in relation to nutritional state and subsequent diet choice in June 2012. These weta had previously been collected from the wild at an earlier instar and kept in captivity for a minimum of three months. Weta were assigned to one of three groups in a two way balanced design that accounted for the variable weights (approximate instars) and sex of the individuals. Under this regime, each individual was housed individually, as described above, but maintained in a temperature controlled room at 15°C with a 14:8 light: dark photoregime throughout the experiment.

Each group was randomly assigned to one of three diets for eight days (phase one of the experiment). The first group was provided with two dishes of the protein rich food (diet P as above; n= 12), the second group with two dishes of the carbohydrate rich food (diet C; n=12), and the third group, which acted as a control (diet CP, n=10), was provided with one dish of each of the foods, allowing individuals to self-select the macronutrient balance in their diet. All dishes initially contained 1 g of food, and were changed every two days. Water was provided *ad libitum*. Dry weights were recorded for the foods before presentation to the weta and after the change two days later, from which amount of protein and carbohydrate ingested were calculated for each weta.

After eight days, the foods were removed from each container. Half of each group was then assigned to a 48 h experimental treatment with one dish of carbohydrate rich food and one dish of protein rich food (phase two). Each weta from the other half of each group was supplied for 48 h with two fresh leaves from the same source plant (mahoe; *Melicytus ramiflorus*) and a dead, thawed tree weta that had been frozen at time of death. We recorded the weight of the dead weta initially and again after 48 h, as well as specific body parts cannibalised, to determine the extent of cannibalism on the dead tree weta. Leaves were digitally scanned before and after presentation to each individual, and the leaf area that had been eaten was then calculated using CompuEye software [42]. Leaf areas were converted into dry weights using specific leaf area data for mahoe (D. Loughlin, unpub. data).

### Long-term nutritional state and cannibalism

For this experiment 28 *Hemideina crassidens* tree weta were kept individually in captivity for a 6 week period and assigned to one of two treatment groups: either a leaf only (low protein) or leaf and protein (leaves and a protein supplement) diet (n = 14 weta in each group). Individuals were fed ad libitum with fresh leaves (*Coprosma robusta* and *Melicytus ramiflorus*) supplied weekly. Captive weta on the protein diet were fed weekly with three Red 8 Protein plus pellets, with a composition of 80% protein, in addition to the leaves. *C.robusta* leaves consist of approximately12.6% soluble carbohydrates and 10.1 % protein, and *Melicytus ramiflorus* approximately 5% soluble carbohydrates and 16% protein (Wehi, Raubenheimer and Morgan-Richards, unpub. data); *M. ramiflorus* is also reported to have an estimated C:N ratio of 29 [43]. Both these species are browsed by tree weta in the wild. Tree weta were maintained throughout in a 15°C temperature controlled room with a 14:8 light:dark photoregime.

After six weeks, all food was removed from the container. Each tree weta was then supplied for 24 h with two fresh, approximately same-sized leaves from the same source plant (*Melicytus ramiflorus*) and a dead, thawed tree weta that had been frozen at the time of death. We recorded all evidence of feeding (herbivory and cannibalism) during this trial period.

### Data analysis

Statistical analyses were carried out in IBM SPSS Statistics version 21. Non-parametric tests were used to ensure robustness and avoid the problems of uncertain distributions. To test for active nutrient regulation, the ratio of protein: carbohydrate eaten was compared to a null model of 1, expected if the weta fed randomly from the high and low protein foods. Medians tests were used to compare treatment groups and sexes. We used chi-squared statistics to examine differences in rates of cannibalism between individuals in the low and high protein groups of the long term nutritional state trial. 

## Results

### Nutrient intake trial

**Figure 1:**
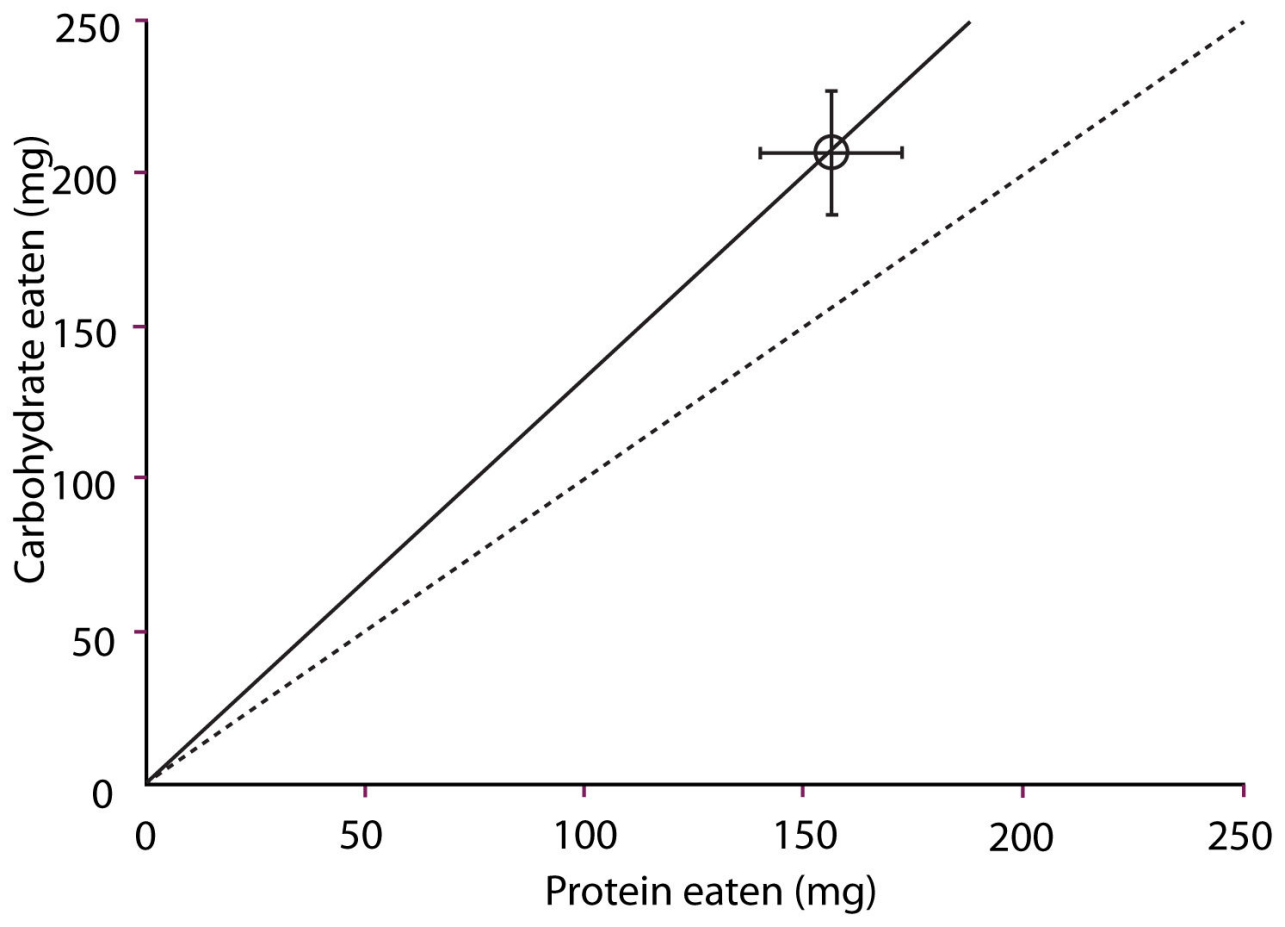
Mean amount of artificial food eaten by Hemideina crassidens individuals over a seven night experimental trial period (x ± SE). Weta selected foods non-randomly so that carbohydrate consumption was greater than protein on average. Dotted line indicates a 1:1 ratio.

**Figure 2:**
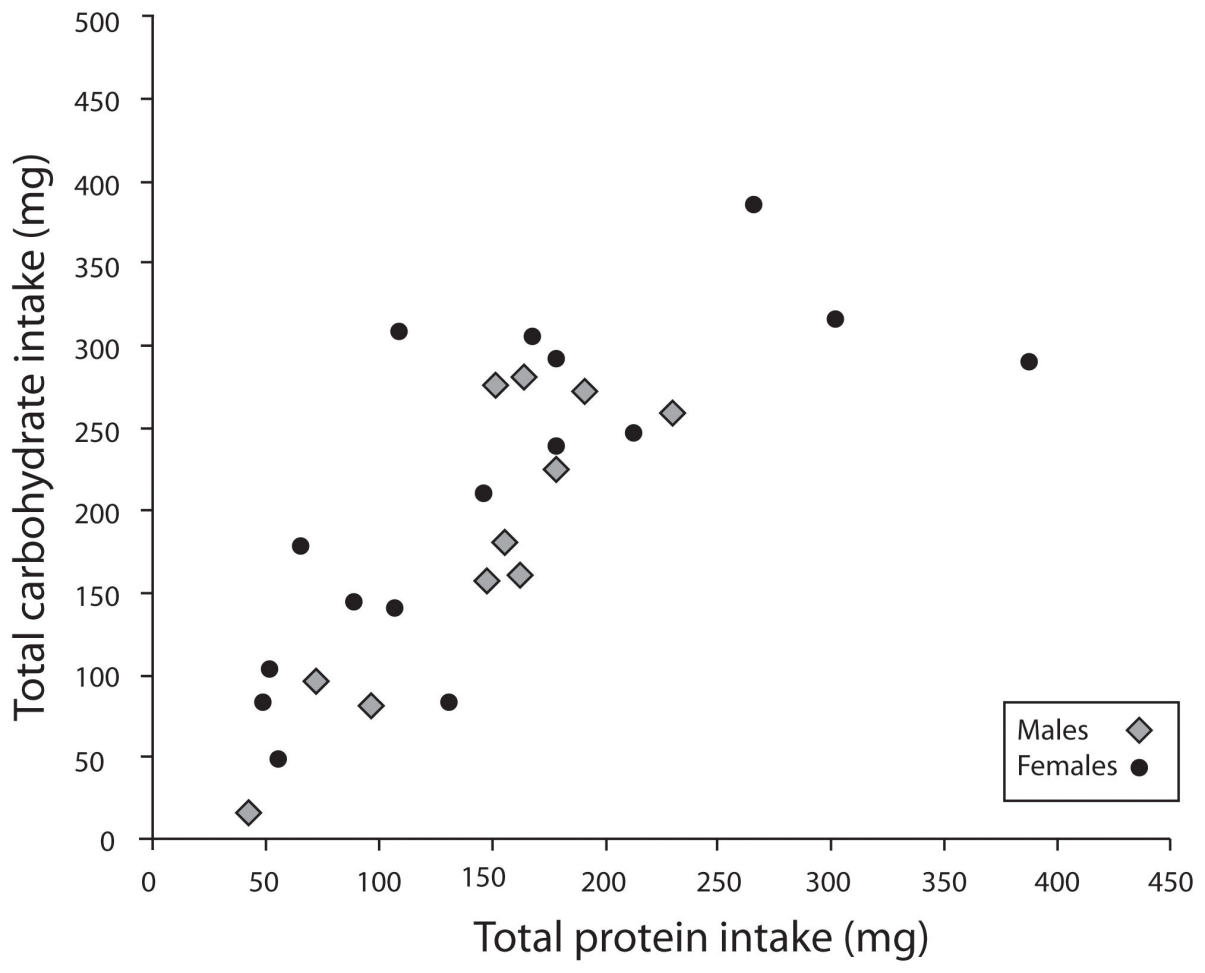
Carbohydrate and protein intake in individual male and female Hemideina crassidens, shown by sex, over a seven night experimental trial period after weta were captured from the wild.

Twenty seven *Hemideina crassidens* individuals completed the feeding trial (n = 17 females and n = 10 males). The ratio of carbohydrate:protein in the selected diet was significantly greater than 1 (Wilcoxon One-sample Signed-rank Test, p=0.009; Figure 1). There was no evidence, however, that this differed between the sexes (Independent-samples Mann-Whitney U test, p=0.12; Figure 2). The data thus indicate non-random selection of the test foods by both male and female weta, to a protein:carbohydrate intake target of 0.84. 

### Influence of Nutritional State on Subsequent Diet Choice

**Figure 3:**
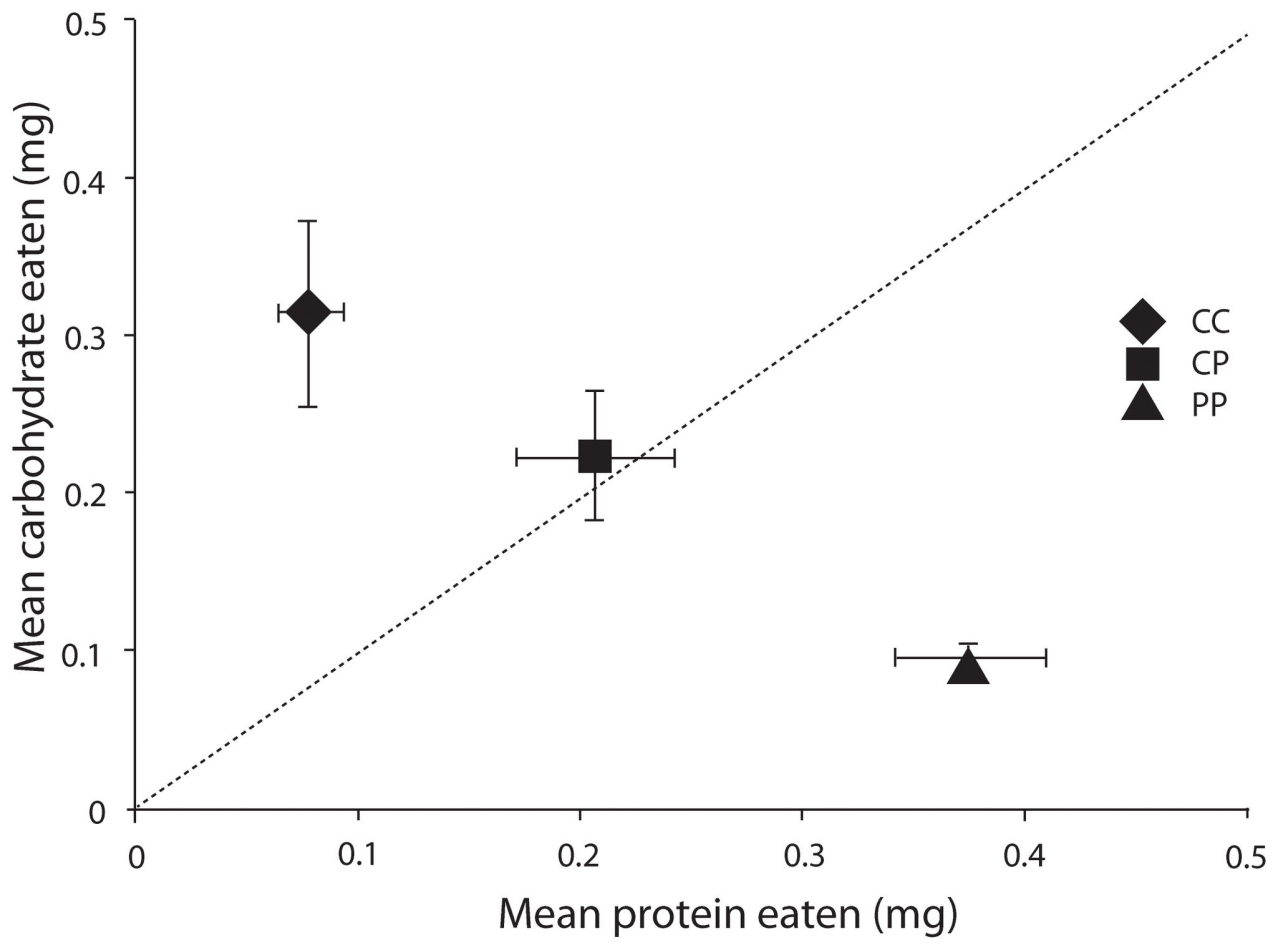
Feeding in the three groups in the eight-day phase 1 of the experiment. Middle line indicates the model if weta were eating the same amount of P-rich and C-rich diet in the mixing treatment (CP). CC indicates the group offered carbohydrate rich food only, CP the group offered both carbohydrate rich and protein rich food, and PP the group offered protein rich food only.

In the first phase of this experiment (eight days), tree weta in the control group (i.e. the CP group, which was able to self-select the macronutrient composition of their diet) selected a protein:carbohydrate ratio that was neither significantly different from the target ratio established in the initial experiment with wild weta, nor from the 1:1 ratio expected if weta were eating equal amounts of the two foods (One-sample Wilcoxon Signed Rank Test, p = 0.445 and p = 0.647, respectively; Figure 3). 

**Figure 4:**
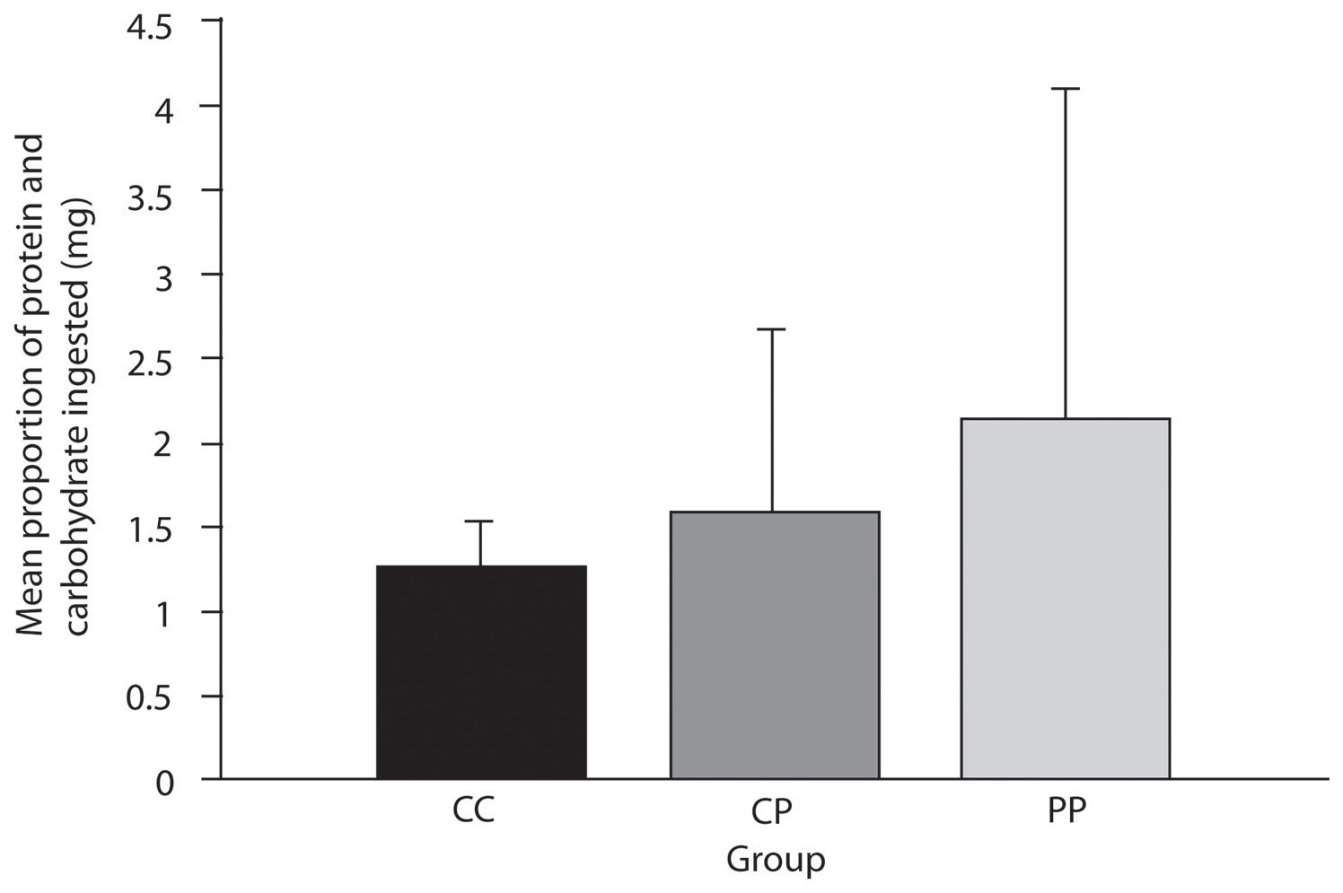
Proportions of carbohydrate and protein ingested by weta during the 48 hour period in phase two. Data are presented according to the previous treatment group of weta, who had been fed for eight days on specific foods in phase 1. CC= carbohydrate rich food only, CP = carbohydrate and protein rich food, PP = protein rich food only.

In the second phase of the experiment, the weta that were maintained on the CP treatment selected a diet with a carbohydrate:protein ratio that was significantly greater than 1 (One-sample Wilcoxon Signed-rank Test, p = 0.043, Figure 4). There was no significant effect of diet in phase 1 on the macronutrient ratio selected in phase 2, either in a comparison of the groups that were confined to the PP and CC treatments in phase 1 (Independent Samples Median Test, p > 0.99) or in a comparison of all three pre-treatment groups (p = 0.855). This indicates that the weta did not compensate for a prior period of confinement to a diet of either excessive protein or excessive carbohydrate. The self-selected diet of all three pre-treatment groups combined was significantly higher in carbohydrate than protein in phase 2 (median protein:carbohydrate ratio = 0.76, p = 0.004, One-sample Wilcoxon Signed Rank Test).

**Figure 5:**
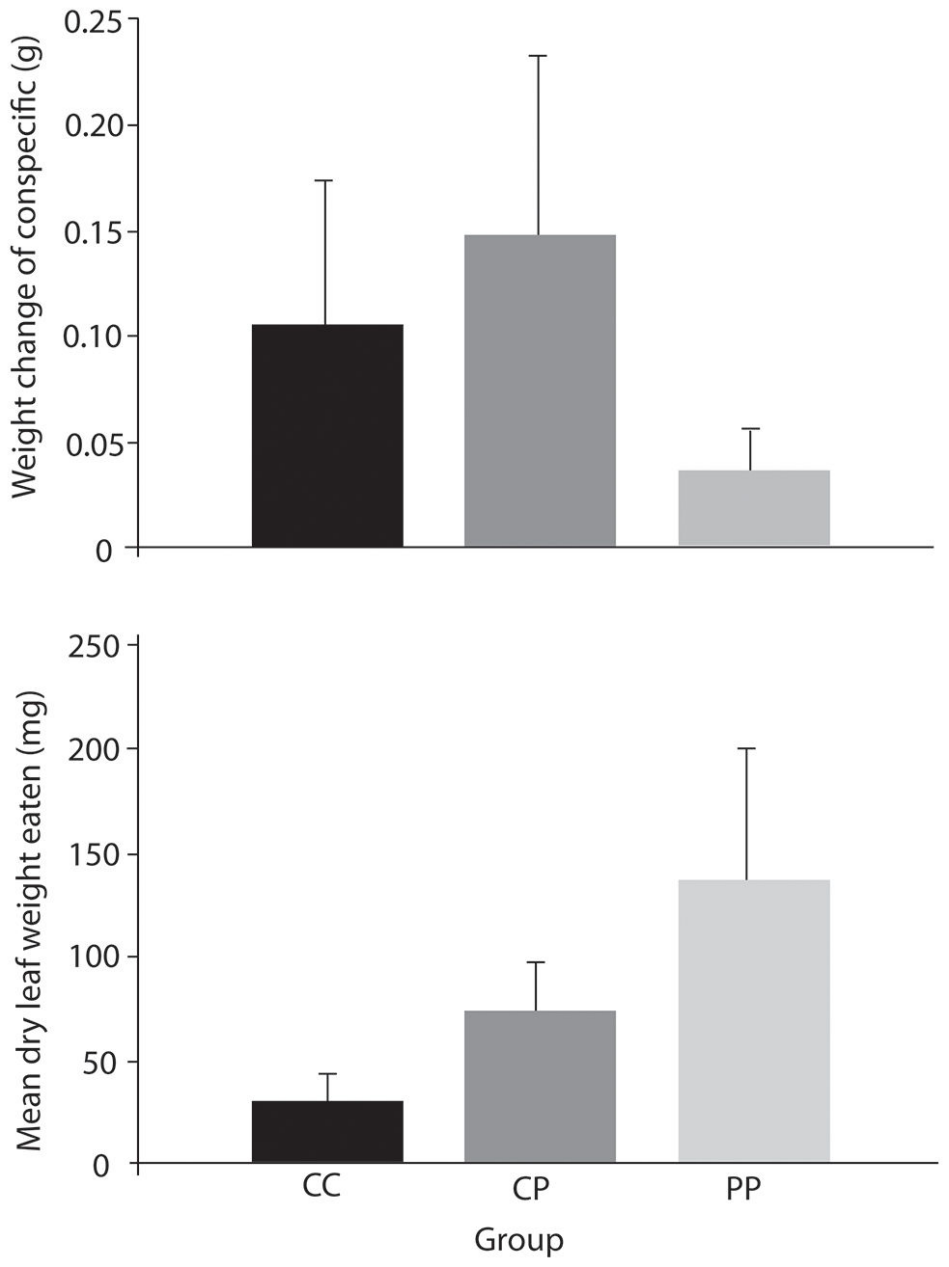
Estimates for the amount of (a) dead conspecific cannibalised and (b) leaf material (dry weight, mg) eaten by tree weta during phase two of the nutritional state trial. Weta had previously been assigned to one of three diets for 8 days. Group CC had carbohydrate rich food only (n=6), CP had a balanced diet (protein and carbohydrate rich foods both available, n=5) and PP, protein rich food only (n=6). Means ± SE presented.

**Figure 6:**
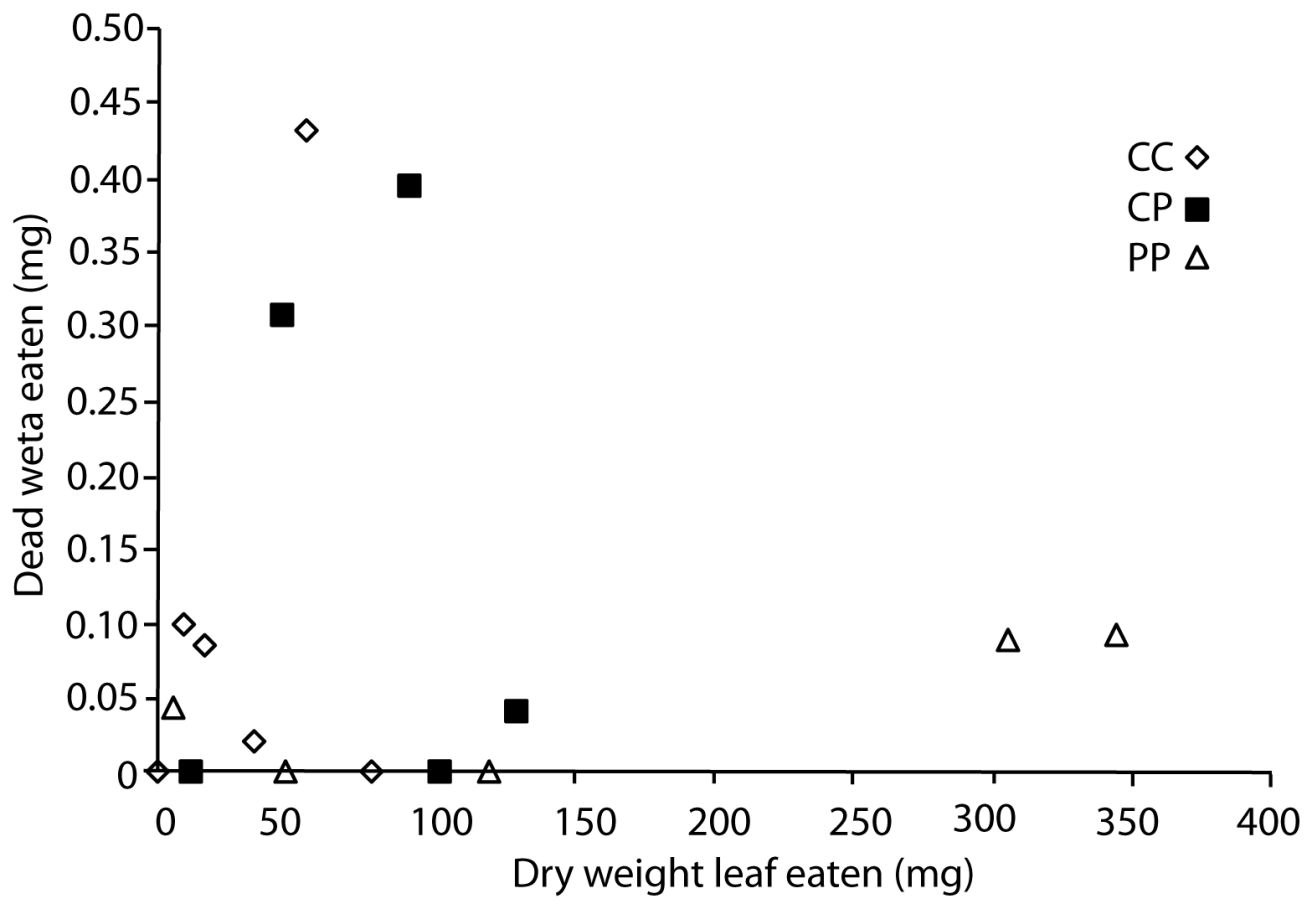
Feeding in the three groups in phase two of the long-term cannibalism experiment. Group CC had previously eaten carbohydrate rich food only for six weeks during phase one of the experiment (n=6), CP had a balanced diet (protein and carbohydrate rich foods both available, n=5) and PP, protein rich food only (n=6).

All tree weta fed leaves and dead conspecifics (rather than the artificial foods) for 48 hours in phase two ate leaf material with the exception of one individual previously in the carbohydrate rich group in phase one. There was no relationship between the amount of flesh eaten (estimated using the weight change of the dead conspecific weta) and previous diet (Independent Samples Median Test, p > 0.93; Figure 5a). Individual weta in the carbohydrate rich group appeared to compensate by eating greater amounts of flesh than leaf material compared to weta in the protein rich group (Figure 6), but a number of weta in the trial ate very little. Feeding in the control group appeared more similar to the carbohydrate rich group than to the protein rich group. Individuals previously on the protein rich diet ate more leaf material on average, but again there was considerable individual variation in the amount of leaf material eaten within each group (Figure 5b). Differences between groups in the amount of leaf material eaten were not significant (Independent Samples Median Test, p > 0.67). 

### Long-term nutritional state and cannibalism

Significantly more tree weta in the protein poor group had cannibalised the dead weta food compared to individuals in the protein rich group during the 24 h trial after six weeks on a protein rich or protein poor diet (X^2^ test, df = 3, p=0.02). Consumption of leaves was universal and thus not strongly influenced by nutritional state.

## Discussion

Many species of animals have been demonstrated to selectively forage based on the macronutrient content of available foods [25]. Resource acquisition can thus be understood by comparing the balance of nutrients available in food to that required by an organism’s phenotype [39,44,45]. Protein and carbohydrate are among the nutrient groups most strongly regulated by herbivorous insects, and are thus expected to play a dominant role in ingestive behaviour [46,47]. We identified a nutrient target for the Wellington tree weta, *Hemideina crassidens*, where individuals select more carbohydrate relative to protein. However, we did not detect a difference between male and female nutrient optima despite strong sexual dimorphism in adults. Although we detected a positive relationship between weta weight and quantity of food consumed, their nutrient target did not change with weight (and, by inference, instar and investment in mandibular weaponry). 

Male tree weta guard tree cavities inhabited by harems of females during the mating season, and expend energy fighting other males for access to these females, using their mandibular weaponry [48]. Male combat can result in significant injury to combatants; male reproductive strategies in highly polygynous species often appear to involve somatic damage and increased viability costs [22,49]. We had therefore hypothesised that substantial dietary costs could be associated with either performance or somatic maintenance in mature males, or that late instar males might require higher protein diets than females during the period when their mandibles develop. However, our data did not show any difference between the sexes in nutrient ratios. The lack of sex-specific differences in nutrient intake probably reflects an equal net reproductive cost for the two sexes, as observed in other species [49]. 

It has been argued that natural selection should favour herbivores that regulate nutrient intake, using pre and post ingestive mechanisms [6]. In the first experiment, after a three day period of starvation weta ate towards a nutrient target that favoured carbohydrate ingestion. In the first phase of the second experiment the group of weta given a combination of complementary foods selected an intake that was not significantly different from the carbohydrate-biased target selected in experiment 1, but was neither different from the 1:1 expected under random feeding. However, in the second phase of that experiment the intake point selected by the same treatment group of weta was significantly higher in carbohydrate than protein, again demonstrating non-random feeding. Together these results show that *Hemideina crassidens* do select their food to reach a carbohydrate:protein intake target that is significantly, if marginally, greater than 1:1. 

 The importance of nutrient balance for fitness would lead us to expect compensatory intake responses after a period of restriction to an imbalanced diet. However, *Hemideina crassidens* did not show a tightly regulated protein response in its patterns of food selection, in contrast to more tightly regulated responses in other insects that have been studied, e.g. [12,50]. As noted above, those that had previously been on a balanced diet ate towards a nutrient target that was weighted towards carbohydrate, supporting the results of the initial experiment. Most individuals were slow to compensate for imbalance through selective nutrient intake. First, tree weta on the protein rich artificial diet did not increase their subsequent (phase two) feeding rate on the carbohydrate rich food available to them. Second, tree weta that had previously been maintained on a carbohydrate rich diet continued to eat a greater amount of carbohydrate rich food than expected in the second phase of the experiment. Third, there was large individual variation in the amount of flesh scavenged by weta in phase two of the eight day intake experiment and no significant differences identified. However, when tested in the long-term trial, a significantly higher number of weta from the protein deprived group cannibalised their dead conspecifics compared to the protein rich group. 

In the wild, individual weta do not forage nightly, and instead favour intermittent feeding based on weather and other environmental conditions (P. Wehi and M. Morgan-Richards, unpub. data). The variability in carbohydrate intake over the seven day period of the initial trial is consistent with a general tolerance to nutrient imbalance. This strategy contrasts with that seen in lab experiments using synthetic foods where tight, target-like, homeostatic regulation of the balance and amounts of nutrients eaten appears to predominate e.g. [12,25]. Nonetheless, non-nutrient components of food, such as indigestible bulk, can also influence food selection [1,47] and nutrient utilisation [51]. It is also possible that if individuals rely on endosymbionts to aid digestion, as has been hypothesised for the Auckland tree weta *Hemideina thoracica* [32], the presence and activity of these endosymbionts could influence both food selection and consumption rate. 

Our results contrast with an extensive literature demonstrating strong short-term compensatory intake responses in a wide range of species [12,13,52,53]. The long term cannibalism trial provides some evidence that weta respond to protein deficit, but this response was weak compared to other Orthopterans such as Mormon crickets, which strongly cannibalise conspecifics when on migratory marches, and when protein deprived in captivity [54]. However, compensatory responses may occur over longer periods than those currently documented with other species, given the wide individual variation in feeding observed in the current experiments. We therefore infer from our data that weta are able to sustain nutrient imbalance for longer periods than other invertebrate species previously used in captive trials. That is, they might have developed adaptive tolerance to imbalance based on intermittent access to high protein sources, e.g. [55,9,56,57], and use this as a strategy to increase fitness in a fluctuating environment.

Overall, these results contrast with other insects that have shown strong and immediate compensatory feeding responses [12,13]. Nonetheless, one difference between our study and most others is that we tested individuals caught directly from the wild. Populations of laboratory organisms are usually inbred (so exhibit little genotypic variation), and through either explicit or implicit selective breeding can show adaptation to laboratory environments including diets. For example, diamondback moth larvae reared in captivity for 350 generations developed fastest on a diet with the same protein/carbohydrate ratio as their lab diet compared with other ratios, and self-selected a diet with the same ratio as their lab diet [58]. However, this same moth species showed adaptation in only 8 generations when fed a high carbohydrate diet [59]. Wild caught individuals, in contrast, can face large variation in the nutrient composition of their naturally occurring diet. In addition, the composition of the population sample is less likely to demonstrate the genotypic and phenotypic uniformity found in lab cultured insects. It is unlikely, however, that our results can be explained entirely by unaccounted variation among field-caught weta. 

Finally, our knowledge of both current and ancestral nutrient environments in the wild remains somewhat limited for tree weta, despite their ecological importance in forest food webs. Future studies that determine heterogeneity in the naturally occurring feeding environment, and diet breadth in the naturally occurring diet of tree weta, will shed further light on the likely strength of selection for nutrient regulation. 

Melissa Griffin and Martin Hanson performed preliminary experiments, and Tracy Harris and Melissa Griffin made up artificial diets. William Wehi and Niwa Wehi helped collect and maintain wild weta. Robyn Dewhurst weighed foods, maintained and fed weta in the second nutrient experiment, and measured leaf area. Daniel Laughlin, University of Waikato, kindly provided data on SLA and LDMCs for mahoe. We thank two anonymous referees for constructive comments on an earlier version of this manuscript. Palmerston North City Council supported the research at Turitea Reserve.
